# γδ T Cells in Autoinflammatory Diseases

**DOI:** 10.3390/cells15050388

**Published:** 2026-02-24

**Authors:** Ilan Bank

**Affiliations:** Rheumatology Unit, Department of Medicine, Sheba Medical Center, Tel Hashomer, Ramat Gan 52621, Israel; ibank@tauex.tau.ac.il

**Keywords:** gammadelta T cells, autoinflammatory diseases, inflammasome, interleukin-1, interleukin-17, familial Mediterranean fever, Behcet’s disease, T cells, interferon gamma, atherosclerosis, Alzheimer’s disease

## Abstract

Autoinflammatory diseases are characterized by inappropriate activation of innate immunity resulting in excessive or persistent inflammation in the absence of infection. γδ T cells possess innate-like properties, including rapid responsiveness to stress-induced self-molecules, phosphoantigens, and inflammasome-derived cytokines, while retaining adaptive effector functions. Neutrophils and macrophages are well-established drivers of autoinflammatory disease, but increasing evidence implicates γδ T cells as key intermediaries by linking innate immune activation to tissue-specific inflammatory pathology. Here, we review evidence that in both monogenic and multifactorial autoinflammatory diseases—including, for example, familial Mediterranean fever, hyper-immunoglobulin (Ig) D syndrome, gout, Behçet’s disease, Still’s disease, atherosclerosis, and neurodegenerative disorders—γδ T cells display altered frequencies, activation states, cytokine polarization, and tissue recruitment. In inflammasome-driven diseases, skewing of γδ T cells toward interleukin (IL)-17 production has been observed, often accompanied by reduced interferon (IFN)γ secretion, thereby amplifying neutrophilic inflammation and tissue damage. In other diseases, e.g., Behcet’s disease, IFNγ and tumor necrosis factor (TNF)α producton predominate. Transcriptomic and tissue-based analyses support the accumulation and functional specialization of γδ T cells at sites of sterile inflammation. Collectively, these findings position γδ T cells as central amplifiers and modulators of inappropriate innate immune activation in the context of autoinflammatory diseases. Improved understanding of γδ T cell subset-specific regulation may inform novel therapeutic strategies targeting autoinflammatory diseases.

## 1. Inappropriate Innate Immunity Activation

Inappropriate activation of innate immunity refers to situations where first-line immune defenses are activated in the absence of appropriate triggers (i.e., infection, noxious chemicals, or adaptive immune responses) or remain activated longer or more potently than required to restore homeostasis. This can cause significant tissue damage and due to acute or chronic inflammation, and thus lead to systemic autoinflammatory diseases. Thus, these diseases are characterized by sterile inflammatory responses resulting from dysregulation of the innate immunity in the absence of a causative intercurrent infectious process or autoimmunity.

Autoinflammatory diseases may be classified according to the causative inflammatory pathway pathogenically involved [1]. The monogenic autoinflammatory disorders are divided into inflammasomopathies, i.e., those caused by mutations in inflammasome components, interleukin (IL)-1-mediated syndromes, tumor necrosis factor (TNF)-related syndromes, interferonopathies, and nuclear factor kappa B (NF-κB) or ubiquitin pathway disorders including the recently described vacuoles, E1 enzyme, X-linked, autoinflammatory, and somatic (VEXAS) syndrome. In addition, deficiency of adenosine deaminase 2 (DADA2) and IL-18-mediated macrophage activation syndrome are also included within this spectrum, as is pyogenic arthritis, pyoderma gangrenosum, and acne (PAPA) syndrome caused by mutations in the *PSTPIP1* gene [2,3,4]. Autoinflammatory diseases, in which no single specific causative mutation has been identified, are defined as polygenic or multifactorial autoinflammatory diseases and include periodic fever syndromes such as periodic fever, aphthous stomatitis, pharyngitis, adenitis syndrome (PFAPA), adult-onset Still’s disease, systemic juvenile idiopathic arthritis (sJIA), neutrophilic diseases [Behçet’s disease (BD), Sweet syndrome, synovitis–acne–pustulosis–hyperostosis–osteitis syndrome (SAPHO), and hydradenitis supporativa], and crystal-induced autoinflammation (gout and pseudogout). Organ-specific autoinflammatory conditions are also multifactorial since they may encompass metabolic, infectious, or autoimmune components, and they include idiopathic recurrent pericarditis, Kawasaki disease (KD), polyarteritis nodosa, and chronic diseases, such as atherosclerosis and neurodegenerative diseases.

## 2. γδ T Cells and the Autoinflammatory Response

The main cellular components of the autoinflammatory response are neutrophils and macrophages. Whereas neutrophils (discovered by Paul Ehrlich in 1879) and macrophages (discovered by Metchnikoff in 1882) are known as prime mediators of inflammation, the role of γδ T cells, discovered more than a century later, in 1986, has only been addressed over the past several decades [5,6]. These cells, which originate from bone marrow-derived precursors developing in the thymus in fetal and post-natal life, express on their cell surface a T cell receptor (TCR) heterodimer encoded by the rearranging γ and δ TCR genes and emerge from the thymus to populate lymphatic and non-lymphatic niches [7]. As opposed to αβ TCRs expressed on conventional cluster of differentiation (CD)4^+^ and CD8^+^ T cells, most γδ T cells do not express CD4 or CD8 co-receptors, a feature commensurate with their ability to recognize antigens directly, in a manner independent of major histocompatibility complex (MHC) molecules, which are required for peptide–antigen presentation to αβ TCRs [8]. γδ T cells comprise 1–10% of all circulating T cells in human peripheral blood (PB), increasing to 30% during certain infections, but they constitute a higher percentage (up to 20%) of intraepithelial T cells in the gut and in the liver at homeostasis [9].

There are two major types of γδ T cells in humans, categorized on the basis of the type of TCR genes expressed on the cell surface. The first, which predominates in the circulation, comprising up to 50–70% of the γδ T cells, expresses the Vγ9 and Vδ2 TCR genes (Vγ9δ2 cells) [9]. The second major subset uses Vδ1 and one of several Vγ genes in the TCR, and it is less numerous in the PB but exhibits marked tissue tropism, predominating in intraepithelial locations (Vδ1^+^ γδ T cells) [9]. A third relatively rare subset uses Vδ3 with various Vγ partners and exhibits features similar to those of the Vδ1^+^ subset [9].

γδ T cells occupy a unique niche in immunology by straddling innate and adaptive immune responses, and their innate reactivities serve as a direct link to autoinflammatory responses. For example, the TCR of the vast majority of Vγ9δ2 cells is selected very early in ontogeny for recognition of phospho-antigens, primarily (E)-4-hydroxy-3-methyl-but-2-enyl pyrophosphate (HMBPP), expressed by pathogens [10]. During infections, HMBPP binds to the intracellular B30.2 domain of BTN3A1, a member of the butyrophilin (BTN) family of cell surface molecules, which is ubiquitously expressed on many types of cells, including antigen-presenting cells (APCs) [11]. This results in the linking of BTN3A1 to BTN2A1 and conformationally perturbed heterodimeric BTN3A1-BTN2A1 complexes on the cell surface membrane [11]. Specific sites of the extracellular domain of this novel structure are recognized by germline conserved motifs of Vγ9 and by complementarity-determining region (CDR)2 of Vδ2 polypeptides of the Vγ9δ2 TCR, driving Vγ9δ2 T cell activation to secrete inflammatory cytokines, primarily IFNγ and TNFα [9]. Thus, the predominant Vγ9δ2 TCR is an innate receptor for a “pathogen associated molecular pattern”, formed by interaction of the conserved pathogen-derived molecule (HMBPP) with BTN3A1-BTN2A1 [12]. Interestingly, isopentenyl pyrophosphate (IPP), an endogenous phosphoantigen, is a product of the mevalonate metabolic pathway in humans. This pathway is upregulated in cells undergoing metabolic stress during malignant transformation or inflammation or by aminobisphosponate drugs. This leads to increased levels of IPP binding to BTN3A1 (albeit with far lower affinity than HMBPP), which induces Vγ9δ2^+^ T cell activation to induce secretion of inflammatory cytokines [13].

The second major set of γδ T cells, i.e., Vδ1^+^ γδ T cells, typically exhibit tissue tropism culminating in epithelial surfaces and express a greater degree of TCR variability and clonal selection, which is dependent upon exposure to pathogens, e.g., cytomegalovirus (CMV) and tuberculosis, suggesting a more “adaptive feature” of this and, similarly, of the Vδ3^+^ and Vδ5^+^ γδ T cell subsets. These subsets recognize cell surface membrane molecules, some of which are induced during cellular stress, including annexin, ephrin A2, endothelial protein C receptor (EPCR), and MHC or MHC-like molecules (CD1d, a, b and c), as well as MHC class I polypeptide-related sequence A (MICA) and MHC class I-related gene protein (MR1), either in their native form or complexed to lipids [8,14]. In these instances, recognition is mediated primarily by the hypervariable CDR3 of Vδ1 chains. Moreover, Vδ1^+^ γδ T cells can recognize shared haptens common to foreign and endogenous proteins [15]. These γδ TCRs may thus exhibit polyspecificity, recognizing multiple ligands of diverse molecular nature, and they are poised to respond very early in an inflammatory reaction that may result in the induction of such Vδ1^+^ γδ TCR ligands [15].

γδ T cells in the periphery that are triggered by antigens binding to the TCR become activated and exhibit a functional repertoire highly reminiscent of classical αβ T cells, including cytotoxicity, cytokine production, and even B cell helper functions [16]. In addition, tissue-localized γδ T cells help maintain and repair damaged tissues by secreting amphiregulin, keratinocyte growth factor, and insulin growth factor (IGF)-1 [17]. The vast majority of human γδ T cells exhibit a T helper (Th)1-like cytokine-producing repertoire, i.e., by the production of IFNγ, but a small yet important proportion may express a Th2-like profile [18]. By virtue of the ability to recognize stress-induced cell surface molecules, γδ T cells can interact with multiple immune and non-immune cell types undergoing stress, and these interactions lead to activation of the appropriate functional programs. For example, interactions with CD14^+^ monocytes with activated Vγ9^+^ γδ T cells may result in the release of inflammatory cytokines, including IL-1β and IFNγ, by the monocytes, which enhances differentiation of macrophages to an M1 phenotype, whereas in adipose tissues, γδ T cells may drive their differentiation to an M2 phenotype [19,20].

**Figure 1:**
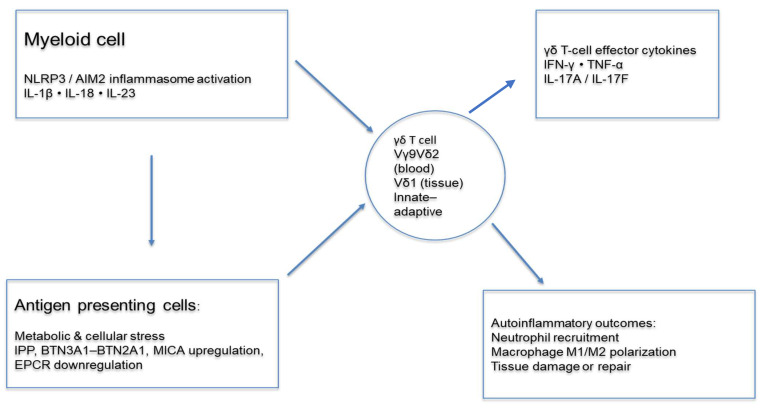
A depiction of how γδ T cells are integrated into the pathogenic mechanisms of autoinflammation is presented. Inappropriate innate immune activation through the NLRP3 and AIM2 inflammasomes in myeloid cells leads to the release of IL-1β and IL-18 and the enhanced production of IL-23, while concomitant metabolic and cellular stress are partly affected by these cytokines, inducing the accumulation of phosphoantigens (IPP), upregulation of BTN3A1–BTN2A1 and stress ligands, such as MICA, and downregulation of endothelial protein C receptor (EPCR) in the cells presenting these antigens. These signals converge on γδ T cells, via TCR-independent (cytokine) or TCR-dependent mechanisms (via recognition of stress-induced molecules), including circulating Vγ9δ2 cells and tissue-resident Vδ1 cells. Activated γδ T cells rapidly produce effector cytokines (IFNγ, TNFα, IL-17A/F), thereby shaping downstream autoinflammatory outcomes, such as neutrophil recruitment, macrophage M1/M2 polarization, and the balance between tissue damage and repair.

Interestingly, in mice, a subset of γδ T cells is pre-programmed in the thymus to emerge as an “innate” IL-17-producing subset [21]. These γδT17 cells undergo “functional pre-commitment” in the thymus, express the canonical Th17 transcription factor retinoic acid receptor-related orphan receptor gamma (RORγ)t, and are also characterized by expression of chemokine receptor 6 (CCR6), IL-23 receptor(R) and IL-1R while lacking expression of CD27 [21]. Importantly, the thymic pre-programming obviates the necessity for TCR engagement for these cells to become activated to produce and secrete IL-17. Rather, they become directly activated by inflammasome-induced cytokines, IL-1β, IL-18, and IL-23, which directly induce production of IL-17A and IL-17F and sometimes granulocyte–macrophage colony-stimulating factor (GM-CSF) and IL-22. Thus, murine γδT17 cells are poised for rapid reactivity to inflammasome activation occurring in response to pathogens but also during inappropriate inflammasome activation, which can occur in autoinflammatory conditions [22]. In humans, it is not clear whether an innate γδT17 subset emerges from the thymus. However, it has been found that human Vγ9δ2^+^ γδ T cells in the periphery can also be activated directly by inflammasome-related products, including IL-18, to produce cytokines [23]. Furthermore, nucleotide-binding oligomerization domain (NOD)-like receptors and pyrin domain-containing 3 (NLRP3) inflammasome have been directly linked to γδT17 cell activation, as demonstrated by the finding that tofacitinib, a Janus kinase (JAK) inhibitor, inhibits excessive NLRP3 inflammasome activation and concomitantly decreases γδT17 cell activation in a model of collagen-induced arthritis [24]. In addition, endocytosed neutrophil extracellular trap (NET)-associated DNA components bind to an intracellular DNA sensor, absent in melanoma 2 (AIM2), which promotes AIM2 inflammasome activation and subsequent gasdermin D-mediated mitochondrial dysfunction, which suppresses the development of regulatory γδ T cells [25]. The nature of γδ TCR reactivity, obviating MHC presentation, functional programs, tissue distribution, and innate reactivity to stress-induced molecules suggests that they could participate not only in appropriate inflammatory reactions, e.g., to external pathogens, but could also be triggered during inappropriate autoinflammation, leading to autoinflammatory diseases, as depicted in Figure 1. Despite the inherent link of γδ T cells to inflammatory responses, the role of these cells has been studied in only a few of the diseases considered to exhibit a prominent autoinflammatory component. Herein, we summarized the currently available data in the scientific literature published in Pubmed, focusing on human diseases, in which the involvement of γδ T cells was addressed. Our major goal was to obtain insights into the potential protective and/or pathogenic contribution of these cells to these diseases, which could suggest new investigative and therapeutic approaches.

## 3. γδ T Cells in Monogenic Autoinflammatory Diseases

An increasing number of diseases characterized by monogenic mutations result in inappropriate inflammatory responses, but γδ T cells have been evaluated in only a few, as described herein.

Familial Mediterranean fever (FMF). This disease results from gain-of-function mutations in the *MEFV* gene on chromosome 16, which codes the pyrin molecule. Whereas in healthy individuals, dephosphorylation of pyrin is not sufficient to provoke its activity, in FMF, dephosphorylation alone drives pyrin binding to the adaptor molecule apoptosis-associated speck-like protein containing a caspase recruitment domain (CARD) (ASC) and to caspase I, driving inflammasome activation and thus unprovoked inflammatory reactions. In this classical autoinflammatory disease, the percentage of PB γδ T cells was not different from that of normal individuals, but the ability of the Vδ2^+^ subset to secrete IFNγ was diminished, and there was a trend to increased number of CCR8^+^ Vδ2^+^ T cells in FMF patients, suggesting increased homing capacity to the sites of inflammation in joints and serous surfaces [26,27].

HyperIgD syndrome (HIDS). This inflammatory syndrome, caused by mutations in the *MVK* gene, which codes for the enzyme mevalonate kinase, is characterized by attacks of chills, arthralgia, myalgia, and abdominal pain, instigated by an increased secretion of IL-1β resulting from defective protein prenylation. γδ T cells in this syndrome have been found to be uniquely defective in their ability to produce TNFα and IFNγ, possibly due to defects in the production of IPP, a downstream product of the mevalonate pathway that triggers the activation of Vγ9δ2 γδ T cells [28].

SAVI (STING-associated vasculopathy with onset in infancy). SAVI is a compound autoimmune and autoinflammatory disease caused by heterozygous gain-of-function mutations of stimulator of interferon genes (STING) [29]. Although no studies of γδ T cells in SAVI have been reported, the characteristic T lymphocytopenia selectively affected the αβ T cell population but not γδ T cells in a murine model of this disease, suggesting their possible involvement in some of the inflammatory manifestations in humans as well [29].

Haploinsufficiency of A20. This disease is caused by either a heterozygous tumor necrosis factor, alpha-induced protein 3 (TNFAIP3) pathogenic variant (~95% of affected individuals) or a heterozygous deletion of 6q23, including TNFAIP3. Patients exhibit febrile episodes, oral or genital ulcers, abdominal pain, and arthritis, thus resembling BD. While no studies of γδ T cells in humans with this disease have been reported, a murine model of *Tnfaip3*^LysM-KO^ mice revealed a reduction in γδ T cells in 18-week-old mice compared to normal mice and spontaneous accumulation of pulmonary Th1, Th17, and γδ17 T cells in *Tnfaip3*^LysM-KO^ *Il17*ra^KO^ mice [30].

Deficiency of adenosine deaminase type 2 (ADA2) (DADA2). DADA2 is a rare inborn error of immunity caused by deleterious biallelic mutations in *ADA2* causing a rare inborn error of immunity with recurrent fevers and vasculitis (ranging from livedo racemosa to polyarteritis nodosa and lacunar stroke), immunodeficiency, and cytopenia, either due to autoimmunity or bone marrow (BM) failure and hematological malignancy. In this disease, the proportions of γδ T cells were similar in healthy donors, heterozygous carriers, and DADA2 patients, but DADA2 patients had significantly fewer Vδ2^+^ cells (as well as other non-conventional T cell subsets) than healthy donors [31].

VEXAS. VEXAS is caused by a reduction in the cytoplasmic UBA1 isoform by somatic *UBA1* mutations, which decreases the efficiency of endoplasmic reticulum (ER)-associated protein degradation. In this disease, characterized by recurrent fever, relapsing polychondritis, vasculitis, pneumonitis, orbital inflammatory syndrome, and Sweet syndrome, γδ T cells exhibited an elevated IFNα and IFNγ gene module score (a quantitative measurement of the overall activity of these genes within a single cell) relative to healthy individuals, revealing their participation in the inflammatory process [32].

The pertinent features relating to γδ T cell perturbations in monogenic autoinflammatory diseases are summarized in Table 1.

## 4. γδ T Cells in Multifactorial/Polygenic Autoinflammatory Disease

### 4.1. Diseases of Inappropriate Autoinflammatory Responses to Well-Defined Endogenous Triggers

Gout. Gout is an autoinflammatory disease induced by inflammasome activation driven by monosodium urate (MSU) crystals, resulting in the release of IL-1β and a downstream inflammatory response involving chiefly neutrophils in joints [33,34]. The NLRP3 inflammasome is a critical component in MSU-induced inflammation. Liu et al. showed that serum IL-17 levels are significantly elevated in the PB early in the onset of symptoms of gout, and they decrease as symptoms diminish [35]. IL-17 expression correlated with serum levels of IL-1β. Flow cytometry analysis indicated that γδ T cells in the PB were the major source of IL-17 production during the early onset of acute gouty arthritis, when approximately 2% of the γδ T cells produced IL-17, reducing to close to 0% when the attack abated [35]. By contrast, the percentages of IFNγ-producing Vδ2^+^ T cells were lower than in normal individuals [27]. Interestingly, moreover, an integrative bioinformatics analysis of the immune cell composition in the joints afflicted by gout revealed significant upregulation of M2 macrophages, activated mast cells, activated NK cells, and γδ T cells [36]. This study further showed upregulation of cell–cell adhesion, leukocyte activation, cell activation, and positive regulation of leukocyte cell–cell adhesion, suggesting that these properties apply to the γδ T cells, and thus they have a pro-inflammatory contribution [36]. An increase in γδ T cells in the joints was likewise reported in a Cibersort analysis of gouty arthritis [37]. These gout samples also exhibited a heightened chemokine (C-C motif) ligand 18 (CCL18), which is a chemokine for adaptive immune cells, but its relevance to γδ T cell migration to joints is unknown [37]. Finally, a study of differentially expressed genes (DEG) relative to normal of primary gout and atherosclerosis patients revealed a number of genes that were significantly upregulated in activated CD4^+^ T cells, γδ T cells, T follicular helper cells, CD56dim natural killer cells, and eosinophils, suggesting that γδ T cells in gout express a unique gene expression profile relative to healthy individuals [38]. Importantly, moreover, the DEGs were mostly enriched in the chemokine signaling pathway, the regulation of actin cytoskeleton, and the TNF signaling pathway, suggesting that, along with other types of immune cells in gout, γδ T cells are pro-inflammatory [38]. In summary, the data strongly reveal involvement of γδ T cells in gout, both in the PB and in the joint space, which is characterized by increased IL-17 and reduced IFNγ secretion by these cells.

Atherosclerosis. Activation of NLRP3 inflammasomes by cholesterol crystals and oxidized low-density lipoproteins (LDL) plays a critical role in atherogenesis, and an inflammatory response is triggered by the NLRP3 inflammasome in foam cells in atherosclerotic plaques, contributing to disease progression and plaque instability [39]. In healthy individuals, there was no prognostic association, between the number of γδ T cells in PB and subsequent occurrence of acute coronary events [40]. Interestingly, however, the frequencies and absolute numbers of total γδ T cells and Vδ2^+^ γδ T cells were found to be significantly decreased in patients with coronary artery disease (CAD) when compared to healthy individuals, and the proportion of Vδ1^+^ T cells was much lower in the patients. However, there was a positive correlation between serum LDL-C levels and frequencies of CD3^+^ γδ T cells, CD69^+^Vδ2^+^ T cells, natural killer group 2D (NKG2D)^+^Vδ2^+^ γδ T cells, and NKp46^+^Vδ2^+^ γδ T cells [41]. Furthermore, γδ T cells in the cardiac tissue during acute myocardial infarction were also depleted relative to normal tissues [42]. Using CIBERSORT and ESTIMATE algorithms, the association between higher LGALS3BP (Galectin-3-binding protein), a glycoprotein that is implicated in inflammation, fibrosis, and cell–cell communication and upregulated in response to cardiovascular stressors associated with adverse cardiac remodeling and immune cell infiltration, was studied. A positive correlation of LGALS3BP with activated dendritic cells, NK cells, memory CD4 T cells, naïve CD4 T cells, CD8 T cells, follicular helper T cells, and γδ T cells in atherosclerotic plaques was revealed, linking γδ T cells to adverse atherosclerosis outcomes [43]. Together, the data suggest that PB Vδ2^+^ T cells may become activated in the presence of LDL, driving their entrance into atherosclerotic plaques and resulting in peripheral depletion. The detrimental contribution of γδ T cells in the context of cardiac atherosclerotic disease is compounded by the finding that higher proportions of γδ T cells in PB were associated with lower absolute (worse) left ventricular global circumferential strain per a 1 SD higher proportion of γδ T cells in a study of older adults [44]. Moreover, in a study to evaluate the link of rheumatoid arthritis (RA) to atherosclerosis, two hub-shared genes, CD52 and tumor necrosis factor receptor superfamily (TNFRSF) 17 genes, were identified. Significant correlations between γδ T cells and TNFRSF17—also known as B cell maturation antigen (BCMA), which is primarily expressed in mature B lymphocytes—in the infiltrate of immune cells, suggest pathogenic interactions of γδ T cells with B cells in atherosclerotic plaques and in RA [45]. Indeed, a separate study confirms increased γδ T cells in atherosclerotic plaques, residing along with M0 macrophages, memory B cells, activated mast cells, and CD4^+^ T cells, which correlates with increased expression of collagen type I alpha 1 (COL1A1) [46]. Atheromatous plaque samples with a high pyroptosis score cluster, as a consequence of inflammasome activation, had higher proportions of γδ T cells, M2 macrophages, myeloid dendritic cells (DCs), and cytotoxic lymphocytes (CTLs) but lower proportions of endothelial cells (ECs) [47]. NOD-like receptor signaling pathways and NFκB signaling pathways were highly enriched in the pyroptosis score high cluster, suggesting a contribution of γδ T cells activated by pyroptotic products in atherosclerotic vessels [47]. Further confirmation of the role of γδ T cells in atherosclerotic plaques was obtained in an analysis of DEGs in atherosclerotic plaques and the correlation of γδ T cells with expression of CD52, a marker present on foam cells [48,49]. Atheromatous plaques in which immune cells were more highly expressed had higher proportions of M0 macrophages and γδ T cells but lower proportions of plasma cells and monocytes (*p* < 0.05). IL18 and other markers were commonly related to these immune cells, suggesting a role for IL-18 in the activation of plaque γδ T cells [50]. Finally, although in mice, IL-23R^+^ γδ T cells are predominantly found in the aortic root, where they promote early atherosclerotic lesion formation, plaque necrosis, and inflammation, a study of human atherosclerotic plaques showed that IL-23 signaling activity was negatively associated with γδ T cells [51,52]. IL-23 signaling may thus be instrumental in inducing elevated levels of αβ and γδ T IL-17A^+^ T cells in the aortas of 21-week-old *Apoe*(-/-) mice fed a Western diet for 15 weeks, but not in humans [53]. Taken together, these studies suggest a model wherein γδ T cells recruited from the PB accumulate in atherosclerotic plaques in response to LDL-C-activated inflammasomes in myeloid-derived foam cells, where they could contribute to local inflammation and plaque instability.

Parkinson’s disease (PD). In this common neurodegenerative disease of motor function, over-activation of the NLRP3 inflammasome in microglia is triggered by α–synuclein that accumulates in PD and indirectly leads to the loss of nigrostriatal dopaminergic neurons [54]. A highly unusual CD4^+^ γδ T cell population secreting IL-17, but not IFNγ, was detected to be significantly increased in the PB of PD patients relative to healthy controls [55]. However, an analysis of the proportions of 22 immune cell types in PB using the CIBERSORT method as well as by flow cytometry revealed that compared with the immune cell proportions in blood samples of healthy control subjects, naïve CD4^+^ T cells and γδ T cells were significantly decreased in PD [56,57]. On the other hand, PB γδ T cells in PD patients were higher than in other neurological diseases, and the γδ T cells in the cerebrospinal fluid more frequently expressed CD25, suggesting an activated state [58]. Thus, the current limited data suggest activation of PB γδ T cells to secrete IL-17 and that γδ T cells in PD may be recruited to the central nervous system and become activated, perhaps by inflammatory products released by α–synuclein-induced activation of inflammasomes.

Alzheimer’s disease (AD). In AD, microglial exposure to pathological amyloid β (Aβ42) and tau peptide aggregates results in an NLRP3 inflammasome-activated pro-inflammatory response [59]. AD patients showed notably elevated proportions of γδ T cells and activated CD4^+^ memory T cells in afflicted brain tissue in comparison to healthy individuals, but not in PB, where they were, however, elevated relative to patients with mild cognitive impairment [60,61,62,63,64,65]. The γδ T cells in the brain exhibited reduced TCR gamma variable (TRGV) 9 clonotypes but were enriched in TRGV2, 4, and 8 clonotypes, which suggests that interactions with BTNL8 expressed in the brain may play a role in the activity of γδ T cells in AD [66]. AD-associated TRG profiles were found in both the PB and brain, and some groups of clonotypes were more specific for the brain or blood in patients with AD compared to those in controls [67]. Recently, it was reported that a higher total cognitive score correlated with a lower expression of genes related to cytotoxicity, antigen presentation, and antimicrobial defense in PB γδ T cells in AD [68,69]. Interestingly, in the murine 3xTg-AD model, an accumulation of IL-17-producing cells, mostly γδ T cells, in the brain and the meninges of female, but not male mice, concomitant with the onset of cognitive decline, suggests early involvement of γδ T cells in the pathogenesis of AD [70].

### 4.2. Diseases of Inappropriate Autoinflammatory Responses to Poorly Defined Endogenous Triggers

Behcet’s disease (BD). Although the underlying causes of this systemic chronic inflammatory disease are complex, BD, a systemic vasculitis with protean clinical manifestations, bears hallmarks of inappropriate inflammatory responses as well as autoimmunity [71]. Triggers of the autoinflammatory response may include bacteria (Streptococcus sanguis), herpes simplex virus, heat shock proteins, environmental cues, and gut dysbiosis [71]. Due to their innate responsiveness to bacterial phosphoantigens, pathogen-related haptens, and stress molecules, γδ T cells appear to be well positioned to respond to these putative triggers of inappropriate inflammation [72]. In fact, γδ T cell response to heat shock proteins was proposed as a useful diagnostic criterion for BD [73]. Furthermore, γδ T cells from BD patients responded to a greater degree to a supernatant of bacterial cultures derived from the oral ulcers of a BD patient [26]. Likewise, an augmented response to Streptococcus sanguis is a specific property of BD CD8^+^ γδ T cells [74]. In active disease, γδ T cells secreted IFNγ but not IL-17A, as opposed to CD4^+^ T cells, which secreted both cytokines but could be triggered to secrete higher levels of both cytokines than normal under IL-17-inducing conditions [75,76]. Other studies suggest that PB γδ T cells in both BD as well as in patients with recurrent aphthous ulcers are increased, express more CD69 and CD29 than healthy controls, and secrete both IFNγ and TNFα [77]. In addition, the Vδ1^+^ subset among CD8^+^ γδ T cells is increased in BD and secretes IFNγ and TNFα [78]. There are conflicting reports with regard to the degree of expansion of γδ T cells in the PB of patients, most consistently indicating expansion during active disease [79,80,81]. At a functional level, it has been found that BD Vγ9δ2^+^ γδ T cells secrete higher levels of granzyme A than normal [82]. Moreover, these cells also expanded fivefold more in patients with active disease than in those with inactive disease or in control individuals in response to the phosphoantigen dimethylallyl pyrophosphate, suggesting inappropriate response to phosphoantigens [83]. One of the major organs afflicted in BD is the eye. High numbers of γδ T cells, predominantly expressing Vγ9δ2 TCR, were detected in the intraocular fluid and responded to phosphoantigens by secreting IFNγ and upregulating CD69 [84]. Likewise, γδ T cells were numerous and observed in all recurrent aphthous lesions of BD patients within the epithelium, inflammatory infiltrates, and at perivascular locations [85]. Taken together, the data indicate an active pathogenic participation of γδ T cells of both major subsets—Vγ9δ2^+^ and Vδ1^+^—in BD, both in the circulating compartment as well as in afflicted organs, by secreting pro-inflammatory cytokines and cytotoxic mediators.

Systemic onset juvenile idiopathic arthritis (Still’s disease, sJIA)**.** Both the percentage and absolute number of γδ T cells increased in active adult Still’s disease [86]. IL-17A was prevalent in sera from patients with Still’s disease, and ex-vivo and in-vitro experiments revealed γδ T cells overexpressing this cytokine. This was not seen with CD4^+^ T cells, which expressed strikingly low levels of IFNγ. Therapeutic IL-1 blockade was associated with partial normalization of both cytokine expression phenotypes. Furthermore, culturing healthy donor γδ T cells in serum from sJIA patients or in medium containing IL-1β, IL-18, and S100A12 induced IL-17 overexpression at levels similar to those observed in the patients’ cells [87]. These data suggest a central role for innate IL-1β- and IL-18-activated and IL-17-producing γδ T cells during active Still’s disease.

Multisystem inflammatory syndrome in children associated with SARS-CoV-2 infection (MIS-C). Although MIS-C is triggered by an external pathogen, its categorization within the scope of autoinflammation resides in the highly exaggerated and pathogenic inflammatory response, suggesting dysregulation of innate immunity, as well as in its clinical similarity to KD. This rare syndrome presents 4–6 weeks after SARS-CoV-2 infection with high fever, organ dysfunction, including cardiac and vascular involvement, and strongly elevated markers of inflammation. The pathogenesis is unclear but has overlapping features with KD as well as likely autoimmune features [88,89]. High levels of IL-1β, IL-6, IL-8, IL-10, IL-17, IFNγ, and differential T and B cell subset lymphopenia are found in the PB during acute disease. High human leukocyte antigen (HLA)-DR expression on γδ and CD4^+^CCR7^+^ T cells is found in the acute phase, and it normalizes at convalescence, suggesting that these immune cell populations are activated and play a role in disease pathogenesis [89]. On the other hand, the number of γδ T cells, along with other T cell subsets, including mucosal-associated invariant T cells (MAIT) and natural killer T (NKT) cells, is reduced, in particular during severe disease [90,91]. Interestingly, 2.2% of MIS-C patients harbor predicted-deleterious variants in BTNL8, which encodes a ligand for Vγ4δ1^+^ γδ T cells in the gut and which were not found in controls. Most of these variants were in the B30.2 domain of BTNL8, a molecule expressed on intestinal epithelium, and the ability of the encoded ligand to activate Vγ4δ1^+^ T cells was impaired. These data suggest a role for functional defects of Vγ4δ1^+^ γδ T cells in the gut, related to the inability to recognize defective BTNL8, in the development of MIS-C-associated enteropathy [92]. In support of this, cells expressing the defective genes exhibited impaired capacity to induce CD69 upregulation and TCR γδ downregulation, indicative of cellular activation [92].

Kawasaki disease (KD). KD, or muco-cutaneous lymph node syndrome, is a form of autoinflammatory vasculitis of medium-sized arteries, including coronary arteries, manifesting with fever, cervical lymphadenopathy, and skin rash. CIBERSORTx-immune cell infiltration analysis revealed that γδ T cells, monocytes, M0 macrophages, activated dendritic cells, activated mast cells, and neutrophils were all elevated in the arterial wall immune infiltrate in KD compared to healthy controls. In patients with coronary artery lesions, there was systemic upregulation of TNFSF13B, C-X-C chemokine ligand 16 (CXCL16), TNFSF10, and interleukin 1 receptor antagonist (IL1RN), mainly produced by monocytes [93]. On the other hand, in the PB, in general, patients with KD had an increasing trend in B cells and monocytes and a reducing trend in CD4^+^ T, CD8^+^ T, MAIT, NK, and γδ T cells compared with controls [94]. A different study showed, however, an increase in γδ T cells in the PB of patients [95]. These results suggest active systemic and local involvement of γδ T cells at sites of inflammation in KD, which may be instigated by the cytokine storm characterizing this disease.

Inflammatory bowel diseases (IBDs). IBDs include several entities—most prominently Crohn’s disease (CrD) and ulcerative colitis (UC). These are pathogenetically highly complex chronic inflammatory diseases with infectious, autoimmune, and autoinflammatory components [96]. While γδ T cells play a prominent role in these diseases, their link to the possible defects of autoinflammatory pathways, e.g., NOD2 and ATG16L1 (Autophagy-Related 16 Like 1), is not well defined [97]. Nevertheless, a general theme appears to be the replacement of homeostatic intraepithelial protective γδ T cells by pro-inflammatory γδ T cells that invade the mucosa. Indeed, recent studies reveal that CD103^+^Vγ4^+^ γδ T cell dysregulation and loss are displayed by humans with germline BTNL3/BTNL8 hypomorphism, and these were identified as a risk factor for penetrating CrD, strongly suggesting a protective role of γδ T cells in this context [66]. Similarly, in UC, it has recently been shown that there are fewer CD103^+^Vγ4δ1^+^ γδ intraepithelial lymphocytes (γδ IELs) and increased γδ T cell subsets with stemlike phenotypes expressing TCF-1 (T cell factor 1) and PD-1 (programmed cell death receptor 1) or effector-like phenotypes expressing granzyme B, perforin, and T-bet in the lamina propria [98]. For detailed analyses of γδ T cell subsets in CrD and UC, the reader is referred to recent reviews on the subject [99,100]. In celiac disease (CeD), classically considered an autoimmune response triggered by gliadin, the alcohol soluble portion of gluten, γδ T cells participate in the exaggerated chronic inflammatory response as well as [101]. Thus, during active CeD, downregulation of BTNL3/BTNL8 on epithelial cells results in loss of the normal Vγ4δ1^+^ γδ T cell intraepithelial population, which bears innate-like cytolytic potential but also expresses genes associated with tissue repair [102,103,104]. These are replaced by Vδ1^+^ T cells enriched in IFNγ-secreting potential upon exposure to gluten, thus contributing to diet-induced inflammation [102,103,104]. Moreover, after gluten ingestion, γδ T cell clones, bearing hallmarks of having been selected by specific antigens, are circulated systemically along with gluten-specific CD4^+^ T cells and assume a cytotoxic phenotype [102,103,104].

A summary of the pertinent features of γδ T cells in the multifactorial/polygenic autoinflammatory diseases is displayed in Table 2.

## 5. Discussion

Autoinflammatory diseases exemplify the pathological consequences of dysregulated innate immune activation, most commonly driven by aberrant inflammasome signaling and excessive production of IL-1 family cytokines [1,2,3,4]. While neutrophils and macrophages are central executors of these responses, γδ T cells appear to function as critical intermediaries that translate innate immune activation into sustained cellular and cytokine-driven inflammation.

A defining feature of γδ T cells is their capacity to respond rapidly to stress signals without the need for classical antigen processing or MHC-restricted presentation [8,10]. This property allows them to sense both exogenous danger signals, such as pathogen-derived phosphoantigens, and endogenous cues, including cell surface molecules and intracellular phosphoantigens generated during metabolic stress and tissue injury, e.g., resulting from crystal deposition [10,11,12,13]. Specific examples include upregulation of MICA molecule by inflammation, which could affect functional outcomes of γδ T cells via interactions with NKG2D, and downregulation of EPCR during inflammation, decreasing interactions of EPCR reactive γδ T cell clones [105,106].

Evidence from monogenic autoinflammatory diseases suggests that γδ T cell dysfunction is not uniform but instead reflects disease-specific alterations in signaling pathways. In FMF, γδ T cell frequencies are preserved, yet Vδ2^+^ cells display impaired IFNγ production and altered chemokine receptor expression, suggesting defective effector function and enhanced tissue homing [25,26]. The driver of the shift toward impaired IFNγ production could be related to the effect of excess IL-1β released due to the genetic defect resulting in inflammasome activation in FMF patients, which would tend to primarily affect putative “innate γδT17 cells” in the circulation. Moreover, recently, it was shown in M694I knock-in (MefvM694I/M694I) mice serving as a model of FMF that Th17 differentiation was enhanced, suggesting that γδT17 subset differentiation might also be affected directly by MEFV mutations in humans [107]. However, given the data indicating tissue homing capacity of the γδ T cells in FMF, which might affect the arthritis and serositis, future studies are required in order to clarify the mechanism of the reduction in IFNγ-secreting γδ T cells and its clinical implications and to examine the possible role of IL-17-secreting γδ T cells in this disease.

In HIDS, γδ T cells exhibit reduced TNFα and IFNγ secretion, likely reflecting impaired production of endogenous phosphoantigens downstream of the mevalonate pathway [27]. Murine models of interferonopathies and NFκB dysregulation further implicate γδ T cells—particularly IL-17- and IFNγ-producing subsets—in shaping inflammatory phenotypes [28,29]. For example, in SAVI, the increased expression of STING ligands could serve to co-stimulate cytokine induction by Vδ2^+^ γδ T cells in the presence of monocytes [108]. Thus, STING ligands strongly stimulated IL-1β and TNFα secretion in monocytes and co-stimulated cytokine induction in short-term expanded Vδ2 γδ T cell lines [108]. In VEXAS, on the other hand, IFNγ profiles increase, demonstrating adaptation of γδ T cells to the immune environment specifically generated in each of the genetically distinct monogenic diseases and reflecting the plasticity of the γδ T cell functional repertoire [32]. A recent study has revealed that mechanistically, the UBA1 defect in VEXAS results in monocyte apoptosis and necroptosis that are strongly enhanced and may be dependent on TNFα and IFNγ, suggesting that the cytokine profile of the γδ T cells in this disease may be contributing to disease pathogenesis [109]. In DADA2, there are no human studies showing γδ T cell involvement, but the pivotal role of innate immunity in the DADA2 pathogenic mechanism is underscored by a skewed polarization from the M2 macrophage subtype to the pro-inflammatory M1 subtype, with an increased production of inflammatory cytokines, such as TNFα, a cytokine highly produced by γδ T cells [110]. Moreover, γδ T cells express receptors for adenosine, which is increased due to the genetic defect of ADA2, and γδ T cells expressing adenosine 2A receptor (A2AR) in vitro generated IL-1, IL-6, IL-17A, and IFNα in response to adenosine [111]. Thus, future studies on the expression and functional repertoire of γδ T cells in DADA2 could be important both for mechanistic and therapeutic understanding.

In the multifactorial autoinflammatory diseases in which defined endogenous triggers have been identified, the contribution of γδ T cells is also apparent, having been studied in more detail. In gout, γδ T cells are a major early source of IL-17 in PB, with cytokine production tightly correlating with IL-1β levels and disease activity [30,31]. Transcriptomic and immune deconvolution analyses further demonstrate enrichment of γδ T cells within inflamed joints and altered γδ T cell gene expression profiles, supporting a direct role in disease pathogenesis [32,33,34]. Together, these findings place γδ T cells downstream of NLRP3 inflammasome activation and upstream of neutrophil-dominated joint inflammation.

A similar paradigm emerges in atherosclerosis, where cholesterol crystals and oxidized LDL activate NLRP3 inflammasomes within foam cells [35,36]. Although circulating γδ T cells—particularly Vδ2^+^ cells—are reduced in CAD, multiple studies demonstrate their accumulation within atherosclerotic plaques, where they associate with macrophages, dendritic cells, pyroptotic signaling, and markers of plaque instability [37,38,39,40,41,42,43,44,45,46]. Correlations between γδ T cell abundance, IL-18 signaling, and adverse cardiac remodeling further support a pathogenic role in vascular inflammation [40,43,46]. Differences between murine and human studies regarding IL-23 signaling and γδT17 cell expansion may be attributed to the unique species-specific nature of γδ T cells, exemplified by the absence of phosphoantigen-reactive murine γδ T cells and the as-yet-unidentified innate γδT17 subset in humans [47,48,49,112].

The involvement of γδ T cells in neurodegenerative diseases with an autoinflammatory component extends the concept that autoinflammation-induced T cells, in addition to those induced by classical immune-mediated mechanisms, may play a role in central nervous system disease. In PD, α-synuclein-induced NLRP3 inflammasome activation in microglia is accompanied by altered γδ T cell frequencies and the emergence of IL-17-producing γδ T cell subsets, both in PB and cerebrospinal fluid [50,51,52,53,54]. In AD, γδ T cells accumulate within affected brain regions and display distinct TCR clonotypes, suggesting ligand-driven selection within the inflamed central nervous system, linking innate and antigen-driven mechanisms of involvement in this disease [55,56,57,58,59,60,61,62,63]. Associations between γδ T cell gene expression signatures and cognitive performance further implicate these cells in disease progression [64,65,66].

Diseases characterized by poorly defined endogenous triggers, including BD, Still’s disease, KD, and MIS-C, provide additional insight into γδ T cell biology in cytokine-rich inflammatory environments. In BD, γδ T cells respond vigorously to microbial products, heat shock proteins, and phosphoantigens, expand during active disease, and secrete pro-inflammatory cytokines and cytotoxic mediators within affected tissues [67,68,69,70,71,72,73,74,75,76,77,78,79,80,81]. In Still’s disease, IL-1β- and IL-18-driven activation of IL-17-producing γδ T cells appears central to systemic inflammation and is partially reversed by IL-1 blockade [82,83]. In MIS-C and KD, immune profiling reveals activation and tissue infiltration of γδ T cells in the context of cytokine storms and vascular inflammation, implicating them in disease pathogenesis despite peripheral lymphopenia [84,85,86,87,88,89]. Moreover, in diseases where autoinflammation as well as autoimmunity have been implicated, such as BD, γδ T cells activated during the inflammatory phase could serve as instigators and regulators of the autoimmune component of the disease by virtue of their ability to present antigens to αβ T cells, activate autoreactive B cells, or act as regulatory T cells [113,114,115,116].

Despite growing evidence linking γδ T cells to autoinflammatory pathology, important gaps remain. γδ T cells have been studied in only a small proportion of autoinflammatory diseases. Moreover, human γδ T cell subsets exhibit marked heterogeneity, and most available data are cross-sectional and derived from PB rather than inflamed tissues. Longitudinal studies, spatially resolved analyses, and functional perturbation experiments in each disease individually will be essential to distinguish precise pathogenic and regulatory roles of γδ T cells in these instances.

From a therapeutic perspective, γδ T cells represent both a challenge and an opportunity. Indirect modulation through inflammasome inhibition, IL-1/IL-18 blockade, or metabolic pathway targeting already shows promise [23,83]. The contribution of IL-17 produced by γδ T cells may explain in part the therapeutic efficacy of anti-IL-17 therapy in gout [117]. The contribution of γδ T cells to TNFα production may be partly responsible for the efficacy of anti-TNFα treatment in BD [118]. A more refined understanding of subset-specific γδ T cell functions may ultimately enable targeted interventions that suppress pathogenic inflammation while preserving tissue-protective immunity.

In summary, γδ T cells emerge as pivotal sensors and amplifiers of inappropriate innate immune activation across a wide range of autoinflammatory diseases. Their ability to integrate inflammasome-derived signals with tissue-specific stress responses positions them at the crossroads of innate and adaptive immunity, with significant implications for disease classification, pathogenesis, and therapy.

## Figures and Tables

**Table 1:** γδ T cells in monogenic autoinflammatory diseases.

Disease	Gene	Dominant Inflammatory Pathway	Key γδ T Cell Insight	Refs.
FMF	*MEFV*	Pyrin inflammasome → IL-1β	Preserved numbers but reduced IFNγ production by Vδ2^+^ T cells; increased CCR8^+^ subset suggests enhanced tissue homing	[25,26]
HIDS	*MVK*	Mevalonate pathway defect → IL-1β	Impaired TNFα and IFNγ secretion due to defective Vγ9δ2 activation (decreased IPP)	[27]
SAVI	*TMEM173*	Constitutive STING → type I IFN	Relative γδ T cell preservation amid αβ lymphopenia suggests participation in IFN-driven inflammation	[28]
HA20	*TNFAIP3*	NFκB dysregulation	Reduced number, but pathogenic γδT17 accumulation in inflamed tissues (murine models)	[29]
DADA2	*ADA2*	Inborn error of immunity	Vδ2^+^ cells reduced	[31]
VEXAS	*UBA1*	Myeloid ubiquitin–stress inflammation	Elevated IFNα and IFNγ gene module scores	[32]

Summary of monogenic autoinflammatory disorders, their causative genes and dominant inflammatory pathways, and the principal quantitative or functional alterations described in γδ T cell subsets, highlighting disease-specific insights into γδ T cell involvement in autoinflammatory pathogenesis.

**Table 2:** γδ T cells in multifactorial/polygenic autoinflammatory diseases.

Disease	Key Trigger/Pathway	Dominant Autoinflammatory Mechanism	γδ T Cell Signature (Key Findings)	References
Gout	Monosodium urate crystals; NLRP3	IL-1β–driven inflammasome activation with neutrophil-mediated joint inflammation	Major early source of IL-17 in blood and joints; reduced IFNγ production by Vδ2^+^ cells	[30,31,32,33,34]
Atherosclerosis	Cholesterol crystals, oxLDL; NLRP3	Foam-cell inflammasome activation, pyroptosis, chronic plaque inflammation	Decreased γδ T cells in blood; accumulation in plaques; associated with IL-18 signaling, macrophages, and plaque instability	[35,36,37,38,39,40,41,42,43,44,45,46,47,48,49]
Parkinson’s disease	α-synuclein; NLRP3	Microglial inflammasome activation and neuroinflammation	Activated IL-17–CD4^+^γδ T cells; variable blood levels; recruitment to CNS and activation	[50,51,52,53,54]
Alzheimer’s disease	Amyloid-β, tau; NLRP3	Chronic microglial inflammasome-driven neuroinflammation	Enrichment in brain tissue; skewed TCR repertoire (decreased TRGV9, increased TRGV2/4/8); IL-17-producing γδ T cells in mouse model early in disease	[55,56,57,58,59,60,61,62,63,64,65,66]
Behçet’s disease	Microbial, stress, and heat-shock antigens	Innate-like immune activation with autoinflammatory and autoimmune features	Expansion and activation of Vγ9δ2^+^ and Vδ1^+^ γδ T cells; secretion of IFNγ, TNFα, granzyme A; prominent tissue infiltration	[67,68,69,70,71,72,73,74,75,76,77,78,79,80,81]
Systemic-onset JIA (Still’s disease)	IL-1β, IL-18	Cytokine-driven innate autoinflammation	Increased circulating γδ T cells with dominant IL-17A production; phenotype partially normalizes with IL-1 blockade	[82,83]
MIS-C (SARS-CoV-2)	Post-infectious immune dysregulation	Systemic cytokine storm with lymphopenia	Numerical reduction but strong activation (HLA-DR^+^); BTNL8 variants impair gut Vγ4δ1^+^ γδ T cell responses	[84,85,86]
Kawasaki disease	Unknown (likely infection-triggered inflammation)	Medium-vessel vasculitis with cytokine storm	γδ T cells enriched in coronary artery infiltrates; peripheral blood findings suggesting redistribution to inflamed tissue	[87,88,89]
Inflammatory bowel diseases (Crohn’s disease, ulcerative colitis) and celiac disease	Microbial dysbiosis; barrier dysfunction; innate sensing pathways (NOD2, ATG16L1); BTNL3/BTNL8	Chronic mucosal autoinflammation with mixed innate and adaptive features	Loss of homeostatic protective CD103^+^ Vγ4^+^ γδ intraepithelial lymphocytes with replacement by pro-inflammatory γδ T cells	[66,96,97,98,99,100,101,102,103,104]

A summary of multifactorial autoinflammatory conditions, their principal triggers and pathogenic pathways, and the characteristic γδ T cell alterations observed in blood and tissues, highlighting shared and disease-specific γδ T cell signatures across crystal-, metabolic-, infection-, and neuroinflammation-driven disorders.

## Data Availability

No new data were created or analyzed in this study. Data sharing is not applicable to this article.

## Abbreviations

**Abbreviation**	**Explanation**
3xTg-AD	Triple-transgenic Alzheimer’s disease
A2AR	Adenosine A2A receptor
AD	Alzheimer’s disease
AIM2	Absent in melanoma 2
APC	Antigen-presenting cell
*Apoe*	Apolipoprotein E
ASC	Apoptosis-associated speck-like protein containing a CARD
ATG16L1	Autophagy-related 16 like 1
BD	Behçet’s disease
BCMA	B cell maturation antigen
BM	Bone marrow
CAD	Coronary artery disease
CARD	Caspase recruitment domain
CCL18	Chemokine (C–C motif) ligand 18
CD	Cluster of differentiation
CDR	Complementarity-determining region
CeD	Celiac disease
CNS	Central nervous system
COL1A1	Collagen type I alpha 1 chain
COVID-19	Coronavirus disease 2019
CrD	Crohn`s disease
CTL	Cytotoxic T lymphocyte
CXCL	Chemokine (C–X–C motif) ligand
DADA2	Deficiency of adenosine deaminase 2
DC	Dendritic cell
EC	Endothelial cell
EPCR	Endothelial protein C receptor
ER	Endoplasmic reticulum
FMF	Familial Mediterranean fever
GM-CSF	Granulocyte–macrophage colony-stimulating factor
HA20	Haploinsufficiency of A20
HIDS	Hyper-IgD syndrome
HLA	Human leukocyte antigen
HMBPP	(E)-4-hydroxy-3-methyl-but-2-enyl pyrophosphate
IFN	Interferon
Ig	Immunoglobulin
IL	Interleukin
IL1RN	Interleukin-1 receptor antagonist
IPP	Isopentenyl pyrophosphate
JAK	Janus kinase
JIA	Juvenile idiopathic arthritis
KD	Kawasaki disease
LDL	Low-density lipoprotein
LDL-C	Low-density lipoprotein cholesterol
LGALS3BP	Galectin-3-binding protein
MAIT	Mucosal-associated invariant T cell
*MEFV*	Mediterranean fever gene (pyrin)
MHC	Major histocompatibility complex
MICA	MHC class I polypeptide-related sequence A
MIS-C	Multisystem inflammatory syndrome in children
M1/M2	Classically vs. alternatively activated macrophages
MR1	MHC class I-related protein 1
MSU	Monosodium urate
*MVK*	Mevalonate kinase
NOD2	Nucleotide-binding oligomerization domain-containing protein 2
NET	Neutrophil extracellular trap
NFκB	Nuclear factor kappa B
NK	Natural killer cell
NKG2D	Natural killer group 2D receptor
NKp46	Natural cytotoxicity receptor 1
NKT	Natural killer T cell
NLRP3	NOD-like receptor family pyrin domain-containing 3
NOD	Nucleotide-binding oligomerization domain
oxLDL	Oxidized low-density lipoprotein
PAPA	Pyogenic arthritis, pyoderma gangrenosum, and acne
PB	Peripheral blood
PD	Parkinson’s disease
PFAPA	Periodic fever, aphthous stomatitis, pharyngitis, and adenitis
*PSTPIP1*	Proline–serine–threonine phosphatase-interacting protein 1
R	Receptor
RA	Rheumatoid arthritis
SAPHO	Synovitis, acne, pustulosis, hyperostosis, and osteitis
SARS-CoV-2	Severe acute respiratory syndrome coronavirus 2
SAVI	STING-associated vasculopathy with onset in infancy
SD	Standard deviation
sJIA	Systemic juvenile idiopathic arthritis
STING	Stimulator of interferon genes
TCR	T cell receptor
Th	T helper cell
*TMEM173*	Transmembrane protein 173 (STING gene)
TNF	Tumor necrosis factor
TNFAIP3	TNF alpha-induced protein 3
TNFRSF17	Tumor necrosis factor receptor superfamily member 17
TNFSF13B	Tumor necrosis factor ligand superfamily member 13B
TRGV	T cell receptor gamma variable
UBA1	Ubiquitin-like modifier activating enzyme 1
VEXAS	Vacuoles, E1 enzyme, X-linked, autoinflammatory, somatic
Vγ/Vδ	Variable regions of γ or δ T cell receptor chains
